# Physical Violence by Partner during Pregnancy and Use of Prenatal Care in Rural India

**DOI:** 10.3329/jhpn.v29i3.7872

**Published:** 2011-06

**Authors:** Alissa D. Koski, Rob Stephenson, Michael R. Koenig

**Affiliations:** ^1^Department of Population, Family and Reproductive Health, Johns Hopkins Bloomberg School of Public Health,615 N Wolfe Street, Baltimore, MD 21205, USA; ^2^Hubert Department of Global Health, Rollins School of Public Health, Emory University, 1518 Clifton Road, Atlanta, GA 30322, USA; ^*^Deceased

**Keywords:** Domestic violence, Pregnancy, Prenatal care, India

## Abstract

The role of physical violence during pregnancy on receipt of prenatal care is poorly understood, particularly for South Asian countries that have high levels of both fertility and domestic violence. Data from the 1998/1999 Indian National Family Health Survey and a 2002/2003 follow-up survey that re-interviewed women in four states were analyzed, examining the association between physical violence during pregnancy and the uptake of prenatal care. Women who experienced physical violence during pregnancy were less likely to receive prenatal care, less likely to receive a home-visit from a health worker for a prenatal check-up, less likely to receive at least three prenatal care visits, and less likely to initiate prenatal care early in the pregnancy. This study highlighted the constraining effect that the experience of physical domestic violence during pregnancy had on the uptake of prenatal care for women in rural India. Maternal health services must recognize the unique needs of women experiencing violence from their intimate partners.

## INTRODUCTION

The prevalence of domestic violence varies widely, with approximately 15-71% of women worldwide experiencing some form of violence at the hands of their husbands or male partners (1). South Asian countries report some of the highest rates of physical domestic violence (2-6). The most recent Indian National Family Health Survey (NFHS 3, conducted in 2005/2006), a nationally-representative survey of women of reproductive age, estimated that 35% of ever-married women had experienced physical violence perpetrated by their current or former spouses (7). In comparison, reported rates of domestic violence, including physical and sexual violence, are consistently lower in countries, such as the USA (1.5%), the UK (4%), and Canada (4%) (1).

A large body of literature describes factors associa-ted with and determinants of domestic violence and the intersection of such violence with negative reproductive health outcomes (6,8-15). However, the vast majority of published work to date has focused on women of reproductive age and has not looked specifically at pregnant women. Of the work that has been published on the effects of domestic violence on pregnant women, most comes from Western nations and uses facility-based data (16-20). There is a particular lack of information on the effects of domestic violence on pregnant women in South Asia where rates of both fertility and domestic violence are high. This paper addresses this gap in knowledge by examining the association between violence during pregnancy and the uptake of prenatal care in India. An understanding of the relationship between violence during pregnancy and the use of maternal health services has the potential to inform the development of health services that recognize the unique healthcare and social support needs of pregnant women who experience domestic violence.

Women may experience violence at any point in their lives. Although there is no conclusive evidence that the risk of domestic violence escalates during pregnancy (21-23), it is clear that a significant subgroup of women are exposed to violence at this vulnerable time (12). Estimates from the USA and other western nations indicate that the prevalence of violence during pregnancy ranges from 0.9% to 20.1% (24). The prevalence of violence during pregnancy among Indian women has been estimated at 18% (13).

Women who experience domestic violence are at an increased risk of many reproductive disorders, such as chronic pelvic pain and sexually transmitted infections (11,25). Their children are also at an increased risk of illness and death (12,24-29). However, evidence suggests that women who experience domestic violence during pregnancy may face a unique set of health issues. Violence can affect pregnancy through direct and indirect mechanisms. Pregnant women are more likely to be struck in the abdomen (30,31), and such blunt trauma may cause adverse outcomes, including foetal injury and death or lead to complications, such as preterm labour (12,18,30-32). Experiencing domestic violence during pregnancy may also have indirect effects on maternal and neonatal health. Psychological stress or restricted access to medical care could potentially lead to poor pregnancy outcomes, such as reduction in birthweight (12,33,34). Evidence from published findings suggests that stress can interfere with the ability of a pregnant woman to obtain adequate nutrition, rest, exercise, and medical care, which may also lead to adverse pregnancy outcomes (30). Stress during pregnancy is also associated with greater likelihood of engaging in negative coping behaviours known to be associated with an increased risk of adverse birth outcomes, such as smoking and substance-abuse (30,35). Women who experience violence during pregnancy are at a significantly greater risk for poor gain in weight and first or second-trimester bleeding and short inter-pregnancy intervals, anaemia, and infections (12,25). These could be the result of diminished ability to control the frequency and timing of sex or to negotiate for use of a contraceptive, particularly methods that require male involvement (11).

One possible mechanism by which violence during pregnancy could be associated with negative pregnancy outcomes is through a constraining effect on women's use of preventative or curative health services during pregnancy (19,36). Studies have found that intimate partners who are physically violent may interfere with the receipt of healthcare by their female counterpart (19,37). One US-based study found that 17% of women who experienced domestic violence also reported that their partners had interfered with their access to healthcare (19). This prevalence is likely underestimated as women in the study were questioned in healthcare settings, thereby excluding those who were completely prevented from accessing care. To our knowledge, no published information is available to document the impact of violence on healthcare-seeking behaviours in South Asian countries.

Results of studies also indicate that women who experience physical violence from an intimate partner are likely to delay the initiation of prenatal care (17,19,36-38). Delay in seeking maternal health services may be due to controlling behaviour by the abuser. Restricted mobility or other controlling behaviours may prevent a pregnant woman from leaving the home or seeking care. Reasons for delaying prenatal care may also include fear of exposing obvious signs of physical abuse, such as black eyes or bruises (19).

Although rates of domestic violence and fertility are high in South Asia, very little information is available to quantify the impact of domestic violence during pregnancy on maternal healthcare-seeking behaviours. This analysis examined the association between experience of physical domestic violence during pregnancy and the uptake of prenatal healthcare in four culturally-contrasting Indian states: Bihar, Jharkhand, Maharashtra, and Tamil Nadu. Standards of living, health indicators, and the status of women vary widely among the four states. Indicators of female autonomy, such as rates of female education, are substantially better in Tamil Nadu (78%) and Maharashtra (76%) than in Bihar (38%) and Jharkhand (41%) (Table 1). Development statistics from the northern states of Bihar and Jharkhand indicate a low standard of living and poor infrastructure (Table 1). Fertility is higher in Bihar [total fertility rate (TFR) 4.0] and Jharkhand (TFR 3.31), where prenatal care is lacking, and indicators of female autonomy are poor. Notably, reported rates of domestic violence are not significantly lower in areas where indicators of female autonomy are better. Reported rates of domestic violence vary from 31% in Maharashtra to 59% in Bihar.

**Table 1.** Selected standard of living and female autonomy indicators by state

Variable	Bihar	Jharkhand	Maharashtra	Tamil Nadu
Female education (%)	38	41	76	78
Male education (%)	72	73	93	91
Household electricity (%)	28	40	84	89
Piped drinking-water (%)	4	11	79	84
Reporting of domestic violence (%)	59	37	31	42
Fertility (TFR)	4.0	3.31	2.1	1.8
Antenatal care (3 or more visits) (%)	17	36	75	97

Source: Indian National Family Health Survey 3, 2005-2006 (7).

TFR=Total fertility rate

This study assessed the effect of physical domestic violence on prenatal care-seeking behaviours in these four states using data collected from the two surveys.

## MATERIALS AND METHODS

Data for this analysis were drawn from a prospective follow-up survey of women who responded to the Indian National Family Health Survey (NFHS) 2 conducted in 1998/1999.

The NFHS 2 sample covered 99% of India's popu-lation residing in all of its states and ultimately included 89,199 women of reproductive age (39). The survey included three sets of questionnaire: (a) household questionnaire which collected sociodemographic information on all household residents; (b) village questionnaire which collected information on the availability of various facilities and services in the village; and (c) women's questionnaire which collected information on women's sociodemographic characteristics, fertility behaviour, familyplanning practices, use of maternal and child healthcare services, general reproductive health, and experience of domestic violence. The overall response rate for sampled women was very high (95.5%) (39).

Data for current analyses were drawn from a pros-pective follow-up survey of original NFHS 2 res-pondents conducted by the International Institute for Population Sciences (IIPS) and the Johns Hopkins Bloomberg School of Public Health in 2002/2003. The sampling frame for the follow-up survey consisted of all respondents interviewed in the original NFHS 2 survey in four Indian states: Bihar, Jharkhand, Maharashtra, and Tamil Nadu. These states were selected to represent differing demographic and socioeconomic contexts in India. The follow-up sample was restricted to rural married women aged 15-39 years at the time of the NFHS 2 interview and who were usual residents of the household at that time. This sample was selected to facilitate the research interests of the survey organizers, which included the relationship between quality of family-planning service and subsequent contraceptive-use and the predictive validity of stated fertility intentions. The follow-up sample included 6,243 women. Re-interview rates were high in all the four states, ranging from 76% in Maharashtra to 94% in Tamil Nadu. A team of 63 female interviewers who had graduate degrees collected data for the follow-up survey. Extensive interview training and field practice were carried out in each state.

The sample used in this analysis was further restricted to women who had at least one birth during the period between the 1998/1999 NFHS 2 survey and the 2002/2003 follow-up survey. This sample included 2,877 women aged 19-43 years.

The survey instrument included questions pertaining to the respondent's background characteristics, reproductive behaviour and intentions, quality of family-planning care, use of family-planning methods and services, an event calendar covering the intervening months between the baseline (NFHS 2) and the follow-up survey (to assess intervening pregnancies, pregnancy outcomes, and monthly contraceptive-use status), prenatal care and immunization, women's status, premarital pregnancyplanning, and domestic violence.

The section of questions on domestic violence explored the respondent's lifetime and recent experience of domestic violence. Following the ethical recommendations of the World Health Organization that only one family member per household be interviewed (40), the youngest eligible woman in the household was selected when multiple res-pondents were available in the household. Specific questions in the survey included the following: “Thinking about your own marriage, has your husband ever: pushed you, pulled you, or held you down? Hit you with his fist or did something that could hurt you? Kicked you or dragged you? Tried to strangle or burn you? Threatened you with a knife, gun, or other weapon? Attacked you with a knife, gun, or other weapon?” The respondents were asked about the number of episodes of each violent act which occurred during the 12 months preceding the survey and the total overall number of episodes of violence which occurred during the preceding 12-month period. In addition, for all women who had one or more pregnancies during the inter-survey period (1998/1999 to 2002/2003), the respondents were asked about the total number of episodes of violence which occurred during their most recent pregnancy.

Four outcomes characterizing the respondent's maternal healthcare-seeking behaviour during her most recent pregnancy were examined in relation to her experience of domestic violence. The four outcomes included: (a) receipt of any prenatal care as indicated by the respondent's answer to a question asking whether or not she went for a prenatal check-up during her most recent pregnancy; (b) receipt of a home-visit from a health worker for a prenatal check-up; (c) receipt of three or more prenatal care visits throughout the duration of the most recent pregnancy as recommended by the Indian Reproductive and Child Health Programme; and (d) the trimester in which prenatal care was initiated. Receipt of prenatal care was modelled for the full sample of 2,877 women while the remaining three outcomes were modelled for a subsample of 1,340 women who received any measure of prenatal care during their most recent pregnancy.

The exposure variable of interest for this analysis was the report of physical violence during the most recent pregnancy. All women who responded to the follow-up survey were asked to estimate the number of violent episodes that occurred during their most recent pregnancy. However, the number of women who reported more than one episode of violence was small enough to prevent the estimation of an odds ratio based on count data. Therefore, the exposure variable used in this analysis is binary and coded as positive for women who experienced any violent act asked about on the survey on at least one occasion during her most recent pregnancy.

Statistical models were fitted for each of the four outcomes. Logistic regression models were fitted to the three binary outcomes of interest (receipt of prenatal care, receipt of a home-based prenatal check-up from a trained health worker, and receipt of at least three prenatal care visits). A multi-nomial regression model was fitted to the categorical outcome (trimester in which prenatal care was initiated). Each model controlled for demographic and socioeconomic characteristics known to be associated with receipt of prenatal care and thought to be potentially confounding in the relationship between violence and prenatal care: age, educational attainment, religious affiliation, ethnicity, employment status of respondents, household standard of living, and parity. Control variables were selected based upon their independent associations with experience of domestic violence and/or receipt of prenatal care. The authors also conducted appropriate tests for interactions among the variables and tests for confounding relationships, none of which proved to be significant. All analyses were conducted using the Stata statistical software (version 9.0).

### Ethical issues

The ethical review committees at the International Institute for Population Sciences and the Johns Hopkins Bloomberg School of Public Health reviewed and approved the follow-up survey protocol and all the survey instruments.

## RESULTS

Table 2 details the distribution of variables considered in this analysis. The sample was largely uneducated (66%) and Hindu (88%) (Table 2). The majority (52%) of the sampled women belonged to backward castes but 58% was employed. Nearly a quarter (23%) of the sample women experienced physical violence during their most recent pregnancy.

**Table 2.** Distribution of variables considered inanalysis of relationship between phy-sical partner violence and receipt of pre-natal care

Demographic variable	No.	%
Experienced physicalviolence during pregnancy		
Yes	657	22.82
No	2,220	77.18
Education of respondent		
No education	1,901	66.06
Primary	531	18.44
Secondary	325	11.31
Higher	120	4.19
Education of partner		
No education	1,010	35.10
Primary	521	18.11
Secondary	992	34.47
Higher	354	12.31
Age (years)		
15-19	388	13.50
20-24	409	14.20
25-29	978	34.00
30-34	716	24.89
35-39	300	10.43
40 and older	86	2.98
Parity		
No previous births	98	3.40
1-2 previous births	841	29.23
3-4 previous births	1,203	41.82
5 or more births	735	25.55
Religion		
Hindu	2,527	87.81
Muslim	274	9.54
Other	76	2.65
Caste		
Scheduled caste	510	17.69
Scheduled tribe	386	13.38
Other backward caste	1,508	52.34
None of them	473	16.39
Employment status of respondents		
Yes	1,680	58.39
No	1,197	41.61

### Impact of physical violence on prenatal care

Women who experienced physical violence during their most recent pregnancy were less likely to receive any measure of prenatal care [odds ratio (OR)=0.80; 95% confidence interval (CI) 0.68-0.95)], less likely to receive a home-based prenatal check-up from a trained health worker (OR=0.43; 95% CI 0.33-0.56), and less likely to receive three or more prenatal care visits (OR=0.66; 95% CI 0.52-0.84) (Table 3). Compared to women who did not experience physical violence, women who experienced one or more violent episodes during their most recent pregnancy were also more likely to receive their first prenatal care visit during the third trimester of pregnancy than during their first trimester (relative risk ratio=1.62; 95% CI 1.08-2.45) (Table 4). Other factors associated with prenatal service uptake were controlled for. The statistical relationship of these sociodemographic characteristics with prenatal care variables is presented in Table 3 and 4.

**Table 3.** Logistic regression models for uptake of prenatal care, receipt of prenatal care from a health worker, and receipt of minimum of three prenatal care visits

Demographic variable	Received any prenatal care (n=2,877)	Received prenatal care from a health worker (n=1,340)	Received minimum of 3 prenatal care visits (n=1,340)
Physical violence during pregnancy (no)			
Experienced violence during most recent pregnancy	0.81 (0.68-0.96)*	0.43 (0.33-0.56)*	0.69 (0.54-0.88)*
Education of respondent (no education)			
Primary	2.29 (1.71-3.07)*	4.75 (3.08-7.31)*	1.74 (1.19-2.55)*
Secondary	3.87 (2.96-5.07)*	4.12 (2.85-5.96)*	2.80 (2.01-3.90)*
Higher	11.52 (5.07-26.22)*	5.87 (3.05-11.28)*	4.31 (2.23-8.32)*
Education of partner (no education)			
Primary	1.93 (1.50-2.47)*	2.66 (1.72-4.10)*	1.29 (0.89-1.87)
Secondary	1.59 (1.29-1.96)*	1.16 (0.78-1.72)	1.08 (0.78-1.49)
Higher	1.54 (1.09-2.18)*	0.53 (0.31-0.93)*	0.96 (0.60-1.52)
Age-group (15-19 years)			
20-24	1.27 (0.74-2.17)	4.97 (1.27-19.42)*	1.04 (0.48-2.25)
25-29	1.76 (1.00-3.08)*	6.87 (1.72-27.35)*	1.50 (0.68-3.31)
30-34	1.75 (0.97-3.16)	7.58 (1.85-31.03)*	1.96 (0.85-4.53)
35-39	1.85 (0.97-3.53)	9.85 (2.21-43.99)*	2.05 (0.80-5.27)
40 and older	1.88 (0.82-4.28)	15.39 (2.22-106.48)*	1.17 (0.32-4.26)
Parity (0 previous)			
1-2 previous births	0.62 (0.48-0.80)*	1.28 (0.90-1.82)	0.98 (0.72-1.34)
3-4 previous births	0.28 (0.21-0.39)*	0.85 (0.51-1.40)	0.85 (0.54-1.33)
5 or more births	0.20 (0.13-0.30)*	0.22 (0.09-0.53)*	0.57 (0.31-1.06)
Religion (Hindu)			
Muslim	0.92 (0.71-1.20)	0.45 (0.25-0.83)*	0.83 (0.55-1.25)
Other	1.67 (0.85-3.23)	2.71 (1.12-6.52)*	2.89 (1.06-7.92)*
Ethnicity (scheduled caste)			
Scheduled tribe	0.56 (0.40-0.80)*	0.31 (0.15-0.62)*	0.68 (0.38-1.20)
Other backward caste	1.21 (0.98-1.49)	0.78 (0.55-1.09)	1.27 (0.93-1.74)
None of them	1.23 (0.92-1.65)	0.17 (0.10-0.27)*	0.70 (0.47-1.03)
Respondent employed at baseline (no)			
Respondent employed at baseline	1.48 (1.22-1.78)*	2.57 (1.91-3.47)*	1.49 (1.14-1.95)*

*Indicates statistical significance at 5% level

Figures are adjusted odds ratios and confidence intervals.

**Table 4.** Multi-nomial regression model for timing of the first prenatal care visit (n=1,340)

Demographic variable	Compared to the first trimester	
	Second trimester	Third trimester
Physical violence during pregnancy (no)		
Experience violence in pregnancy	1.20 (0.94-1.52)	1.62 (1.08-2.45)*
Respondent's educational level (no education)		
Primary	0.81 (0.56-1.18)	1.03 (0.54-1.95)
Secondary	0.58 (0.42-0.80)*	0.74 (0.42-1.32)
Higher	0.54 (0.29-1.00)*	0.25 (0.05-1.20)
Education of partner (no education)		
Primary	1.04 (0.72-1.52)	0.47 (0.25-0.88)*
Secondary	1.10 (0.78-1.53)	0.51 (0.30-0.87)*
Higher	1.07 (0.67-1.71)	0.71 (0.34-1.50)
Age-group (15-19 years)		
20-24	0.49 (0.21-1.14)	0.53 (0.13-2.15)
25-29	0.44 (0.18-1.03)	0.52 (0.12-2.22)
30-34	0.34 (0.14-0.84)*	0.47 (0.11-2.11)
35-39	0.38 (0.14-1.05)	0.56 (0.11-2.84)
40 and older	0.34 (0.09-1.33)	0.26 (0.03-2.35)
Parity (0 previous births)		
1-2 previous births	1.16 (0.85-1.58)	1.75 (0.96-3.22)
3-4 previous births	1.27 (0.81-1.99)	2.52 (1.16-5.46)*
5 or more births	1.79 (0.96-3.35)	4.10 (1.56-10.73)*
Household standard of living (low)		
Medium	0.88 (0.67-1.16)	1.25 (0.80-1.95)
High	0.41 (0.25-0.67)*	0.39 (0.14-1.09)

Figures are hazard ratios and confidence intervals

*Indicates statistical significance at 5% level

## DISCUSSION

Domestic violence represents a significant public-health problem in resource-poor countries. Although progress has been made in documenting the prevalence of domestic violence and examining its contextual determinants, the mechanisms by which such violence affects maternal and child health are not well-understood. Domestic violence may lead to increased perinatal or neonatal morbidity and mortality directly through physical trauma or indirectly through increased stress on the mother during pregnancy (12,33,34). Alternatively, women who experience violence may be exposed to other controlling behaviours that limit their access to healthcare, thereby limiting their ability to seek care for themselves or their children. This analysis provides support for the latter as a mechanism through which domestic violence may have adverse effects on pregnancy outcomes and also on maternal and child health. These findings indicate that prevention of domestic violence is likely to contribute to further reductions in maternal and early childhood morbidity and mortality.

The reported prevalence (23%) of physical violence during pregnancy in this sample is substantially higher than estimates of exposure to physical violence during pregnancy in other parts of the world (41-44). However, a few studies in India have estimated similar rates of violence during pregnancy. A large-scale survey of married women with at least one child conducted in seven sites throughout India estimated that 28% of respondents had experienced psychological and/or physical violence during pregnancy (45). Another smaller-scale study estimated that 22% of women interviewed during the third trimester of their pregnancy had experienced physical violence during their current pregnancy (46). Interviews in the second study were conducted at prenatal care clinics, which excluded women who did not receive facility-based care. This makes it likely that the prevalence of violence was an underestimate. A third study estimated the prevalence of domestic violence during pregnancy in Uttar Pradesh, India, to be 18% (13). The rate estimated in a single state is difficult to compare with this analysis which includes data from four states that vary widely in terms of women's autonomy and development statistics. The small number of other estimates with which to compare the prevalence reported in this sample highlights the need for additional research on this public-health issue, particularly in South Asia where levels of domestic violence and fertility are high. More generally, the strikingly high rates reported in India compared to the rest of the world are cause for concern. A greater understanding of the reasons for these high rates is needed.

The results of this analysis indicate a strong relationship between the experience of physical violence during pregnancy and constrained maternal healthcare-seeking behaviour. Compared to women who did not experience violence, women in this analysis, who experienced at least one violent episode during their most recent pregnancy were 20% less likely to receive prenatal care and nearly 60% less likely to receive a home-based prenatal check-up from a trained health professional. They were also less likely to receive three or more prenatal care visits and more likely to begin prenatal care late in pregnancy. Decreased access to and/or use of maternal health services during pregnancy may prohibit women from obtaining necessary preventive or curative treatment that could improve their health and the health of their children.

Among the outcomes examined in this analysis, the greatest disparity between women who experienced physical domestic violence during pregnancy and those who did not was observed in the receipt of home-based prenatal check-ups from health workers. Only 26% of women who experienced violence during pregnancy received a prenatal care visit from a health worker at their home compared to 50% of women who did not report violence. There are a number of possible explanations for this discrepancy. Lack of mobility due to controlling behaviour from a partner may limit the involvement of a woman in the community, thereby decreasing the likelihood that local health workers would be aware of her pregnancy. Even if the pregnancy were recognized, a partner's controlling behaviour may limit the ability of a health worker to visit the woman at home. Although domestic violence is recognized as a criminal offence in India, this widespread problem is largely tolerated. Reporting violence, or discussing it with a health worker, may lead to more violence. Finally, uneducated women and women belonging to scheduled tribes were especially unlikely to receive a prenatal check-up at home from a health worker, indicating that social structures and community-level factors may also affect service-provision patterns.

Despite this difference in type of service, women who experienced violence during pregnancy received prenatal care at a rate similar to women who did not report violence. Forty-four percent of women who reported violence received some measure of prenatal care as did 50% of women who did not report violence. This suggests that women who experience violence during pregnancy do not abandon maternal healthcare altogether but may seek or receive care from alternate sources. For example, women who experience domestic violence may be more likely to seek prenatal care from untrained providers, such as traditional birth attendants or family members. Additional research is needed to better understand how women who experience violence during pregnancy seek prenatal care.

Many studies have focused on the inclusion of violence screening during provision of maternal health services (47,48). The evidence provided here makes it clear that women who experience violence during pregnancy are less likely to receive prenatal care, and therefore, delaying violence screening until this point may miss a significant proportion of women it is intended to catch. The WHO recommends that providers of family-planning service integrate questions regarding domestic violence into routine history-taking and offer emotional support to those experiencing domestic violence (49). Initiating conversations about violence before pregnancy, whether at family-planning service visits or routine health visits by women is likely to detect a greater percentage of women who are at a risk of experiencing violence during pregnancy.

### Limitations

Two potential limitations of this study should be noted. The first limitation of this analysis is our inability to know the exact temporal relationship between violence and prenatal care during pregnancy. From these data, we are unable to determine whether the violence occurred before or after prenatal care described. However, this study represents an improvement over previous work as we know that the violence and prenatal care reported were occurring within the same pregnancy. The second limitation concerns our reliance upon self-reports of domestic violence. The high level of social acceptance and limited repercussions for perpetrators of domestic violence in this setting (2) lead us to believe that under-reporting of domestic violence and resultant measurement error is not likely to be of sufficient magnitude to compromise the validity of our findings. In addition, the levels of violence reported in the NFHS 2 report are validated by another population-based study in which a similar proportion of Indian women experienced violence (50).

### Conclusions

Given the undeniable impact of domestic violence as a public-health problem in this setting, understanding the relationship between experience of violence and negative health outcomes is critical for the development of appropriate prevention strategies and maternal health services that are responsive to the needs of women experiencing violence. Levels of maternal and child morbidity and mortality remain high in India despite decades of programmatic efforts to improve the health of these populations. This analysis provides support for continued efforts to reduce levels of domestic violence through channels such as public education, legal reform, and community action, as a means to reducing morbidity and mortality among both these groups.

## ACKNOWLEDGEMENTS

The study was funded by the National Institute of Child Health and Human Development (Grant No. R01HD039405).
